# Spontaneous rupture of hepatic metastasis from small cell neuroendocrine carcinoma of maxillary sinus

**DOI:** 10.1186/1477-7819-12-126

**Published:** 2014-04-27

**Authors:** Yun-fei Duan, Yan Tan, Bo Yuan, Feng Zhu

**Affiliations:** 1 Department of Hepatobiliary Surgery, The Third Affiliated Hospital of Soochow University, 185 Juqian Street, Changzhou, Jiangsu 213003, China; 2 Department of Pathology, The Third Affiliated Hospital of Soochow University, Changzhou 213003, China; 3 Department of Hepatobiliary Surgery, Changzhou TCM Hospital, Changzhou 213003, China

**Keywords:** Maxillary sinus, Small cell neuroendocrine carcinoma, Hepatic metastases, Hemoperitoneum, Hepatectomy

## Abstract

**Background:**

Small cell neuroendocrine carcinoma of the maxillary sinus, a rare malignant tumor, has a poor prognosis because of its high incidence of metastasis. Moreover, metastatic cancer-induced hepatic rupture, characterized by hemoperitoneum, is infrequent, although several lines of evidences have reported that a wide variety of other neoplasms can cause this usually fatal manifestation.

**Case presentation:**

We now present the first case of a 49-year-old man with spontaneous rupture of hepatic metastasis from small cell neuroendocrine carcinoma of the maxillary sinus and ultimately resulted in massive intraperitoneal bleeding, which was successfully controlled by subsequent surgery (partial hepatectomy). The postoperative clinical manifestation of the patient was uneventful. He was discharged on the 16th day after operation and without any complication.

**Conclusions:**

Small cell neuroendocrine carcinoma of the maxillary sinus is very scarce and unfortunately has a poor prognosis. It has potential to cause spontaneous metastatic rupture which can elicit fatal hemorrhage. Emergency surgery is effective, although the long-term outcome is still unsatisfactory.

## Background

Small cell neuroendocrine carcinoma of maxillary sinus is a rare and aggressive malignant disease. Non-traumatic rupture of the liver frequently occurred in primary benign or malignant tumors, metastatic carcinoma, peliosishepatis, polyarteritisnodosa, systemic lupus erythematosus, and toxemia of pregnancy [1]. However, spontaneous hepatic rupture induced by metastatic cancer, which presented as hemoperitoneum, is not common [2]. We currently reported a patient with hemoperitoneum due to bleeding hepatic metastasis which was originated from small cell neuroendocrine carcinoma of the maxillary sinus, although there is no literature reporting it.

## Case presentation

**Figure 1:**
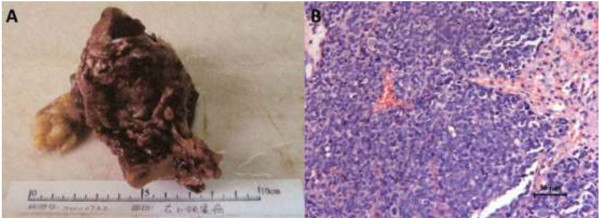
**The specimen of the maxillary sinus carcinoma (A) and it’s HE staining (B).** The tumors are composed of small-sized regular cells which are arranged in broad sheets, nests, and cords**.**

A 49-year-old man had undergone surgery to resect a small cell neuroendocrine carcinoma (SNEC) of the right upper maxillary sinus 21 months previously (Figure 1). He underwent preoperative ‘pingyangmycin’10 days of chemotherapy. The patient complained about persistent and severe right upper quadrant pain which lasted about 6 hours.

We observed that he had a pale complexion and distended, tender abdomen after careful physical examinations. The vital signs were a temperature of 36.8°C, blood pressure of 90/55 mmHg, and pulse rate of 130/min.

**Figure 2:**
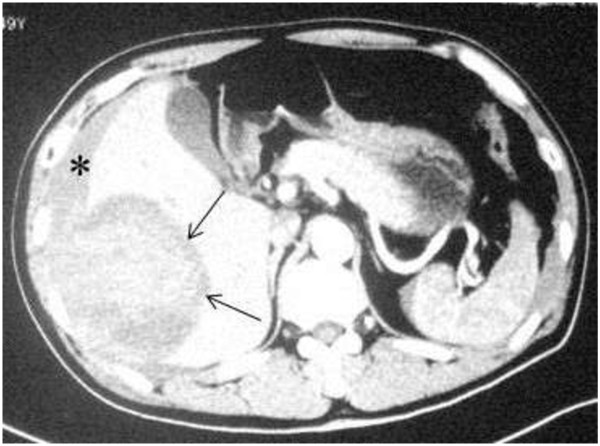
CT reveals a large tumor (arrows) in the right lobe of the liver along with an adjacent intraperitoneal hyperdense fluid collection (black asterisk).

The hematological data were as follows: white blood cell (WBC) count 12.4 × 10^3^/mL (3.5 × 10^3^/mL-9.5 × 10^3^/mL), hemoglobin 80 g/L (130-175 g/L), hematocrit 0.233 (0.4-0.5), platelets 161 × 10^3^/mL (125 × 10^3^/mL-350 × 10^3^/mL), and prothrombin time (PT) 13.3 s (9-13 s). The hepatic function data were as follows: alanine aminotransferase (ALT) 98 U/L (9-50U/L), aspartate transaminase (AST) 15 U/L (15-40U/L), γ-glutamyltranspeptidase (γ-GTP) 164 U/L (10-60U/L), total protein 56.6 g/L (60-82 g/L), and total bilirubin 9.6 μmol/L (4-19 μmol/L). Markers for hepatitis viruses except for hepatitis B surface antibody were all negative. As to tumor markers, serum levels of carcino-embryonalantigen (CEA) were slightly increased. Additionally, serum levels of carbohydrate-antigen 19-9 (CA19-9), neurone-specific enolase (NSE), alpha-fetoprotein (AFP), cytokeratin 19 fragment (CY211), carbohydrate-antigen 125 (CA125), carbohydrate-antigen 724 (CA724), prostate-specific antigen (PSA), and squamous cell carcinoma (SCC) were totally at normal levels (Table 1). Abdominal ultrasonography showed that isolated lesion corresponded to blood on diagnostic puncture in the liver and ascites. Computed tomography (CT) scan showed a large tumor in the right lobe of the liver and with an adjacent intraperitoneal hyperdense fluid collection (Figure 2). Besides, there was no underlying cirrhosis. Furthermore, CT scan revealed no abnormity in both lungs. We considered that the tumor was resectable which was based on the results of CT scan and liver function.

**Table 1:** Tumor markers of the patient

**Parameter**	**Patient**	**Normal range**
CEA (ng/mL)	6.77	0.00-5.00
CA19-9 (U/mL)	21.71	0.00-37.00
AFP (ng/mL)	3.04	0.00-8.00
NSE (ng/mL)	0.9	0.00-16.30
CA125 (U/mL)	9.60	0.00-35.00
CA724 (U/mL)	0.2	0.00-6.90
CY211 (ng/mL)	3.16	0.10-3.30
PSA (ng/mL)	0.56	0.00-4.00
SCC (ng/mL)	0.1	0.00-1.50

After fast-track resuscitation (4 units of packed red blood cells (600 mL) and 1,000 mL hetastarch 130/0.4 hydroxyethyl starch), the hemodynamics of the patient became relatively stable. Moreover, 2,500 mL of blood with clots were observed in the abdominal cavity in the operation. The liver tissue of the patient showed a normal color and a smooth surface in appearance, but there was an isolated lesion in segment 6 with a 6 cm laceration of the capsule, which skeptically was malignant tumor from macroscopical observation. There were no other observed bleeding sites. A resection of segment 6 of the liver was performed.

**Figure 3:**
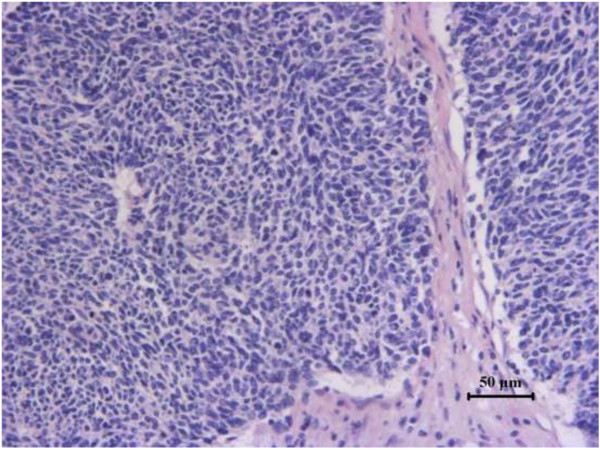
**The hepatic tumors are composed of small-sized regular cells which are arranged in broad sheets, nests, and cords.** Many of the cells contain cribriform nuclei with a fine reticular chromatin pattern and small amounts of cytoplasm.

**Figure 4:**
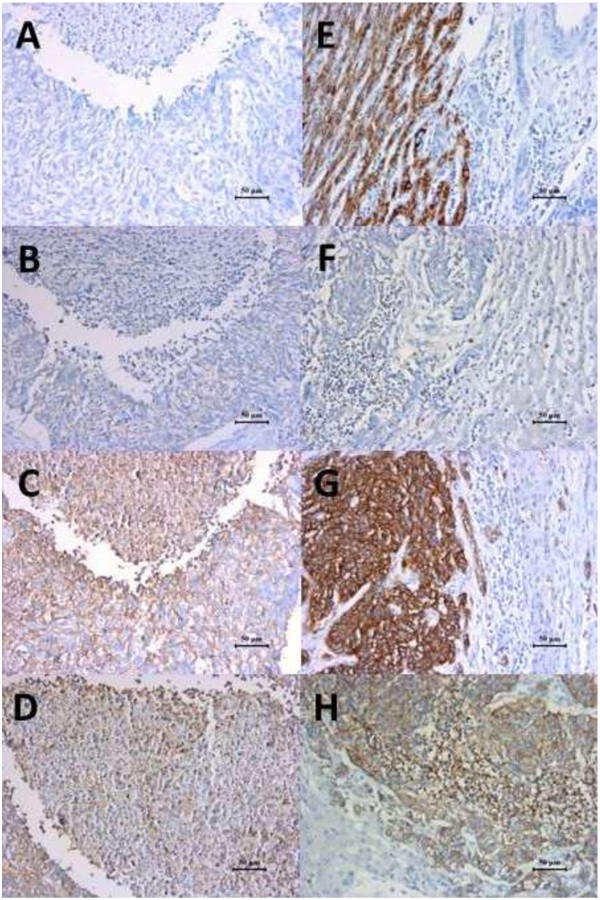
**Maxillary sinus carcinoma and metastatic hepatic carcinoma stained negative for Hepatocyte (A,E) and CgA (B,F), while positive for CD56 (C,G) and Syn (D,H).**

The resected tumor was 8 × 7 × 6.5 cm in size. The immunohistochemisty findings were as follows: Hepatocyte (-), CK5/6 (-), CK34 (-), P63 (-), CK8/18 (+), Vimentin (-), CK35 (+), Ki-67 proliferation index was more than 70% (+). Combined with the observation of HE staining (Figure 3), microscopic examination of the tumor confirmed a metastatic poorly differentiated carcinoma. Repeated immunohistochemistry for tissues of carcinoma of maxillary sinus and metastatic hepatic carcinoma showed that Hepatocyte(-), CgA(-), CD56(+), Syn(+) (Figure 4). Ultimately, we diagnosed and defined as hepatic metastases from small cell neuroendocrine carcinoma of maxillary sinus.

The patient was discharged on the 16th day after surgery and without any complication. He declined chemotherapy and died of cachexia and pulmonary metastasis after 7 months without the incidence of recurrent hemoperitoneum.

## Discussion

Small cell neuroendocrine carcinoma of the sinonasal region is not common of the sinonasal carcinomas with neuroendocrine differentiation [3]. The histogenesis of the neuroendocrine differentiated tumors is not clear. It has been postulated that, outside the lung, this tumor is derived from the neuroendocrine amine precursor uptake decarboxylase (APUD) cells, which are widely distributed in the body. The tumors are composed of small-sized regular cells which are arranged in broad sheets, nests, and cords. Many of the cells contain cribriform nuclei with a fine reticular chromatin pattern and small to moderate amounts of cytoplasm [4]. Immunohistochemical observation is essential to make a pathological diagnosis. SNEC has been reported to stain strongly with synaptophysin and CD56 nerve cell adhesion molecule and weakly with chromogranin A and CAM 5.2/AE-1 [5]. The prognosis in cases of head and neck SNEC is very poor because of its high metastatic rate [6]. In this patient we reported, the tumor metastasized to the liver 21 months after maxillectomy.

Metastatic disease of the liver resulting in spontaneous hepatic rupture is rare as compared with primary hepatic tumor [2,7]. This probably reflects the tendency of metastatic cancer to be less vascular and invasive, and to penetrate the liver capsule less frequently than primary tumor [7]. A recent study reviewed that there have some reported cases of spontaneous rupture of hepatic metastases from primary sites including the prostate, lung, esophagus, stomach, kidney, colon, pancreas, testicle, gallbladder, skin, choriocarcinoma, malignant melanoma, nasopharynx, and others [7-20]. But there was no report regarding rupture of hepatic metastases from SNEC of the maxillary sinus.

The typical presentation is sudden epigastric or right hypochondria pain. Shock could be observed in some patients, while most patients were manifested with peritonitis or abdominal distension. Some patients are paracentesis-positive with blood-stained ascites. Ultrasonography, a simple, quick, non-invasive investigating method, can locate the ruptured tumor and reveal a free intraperitoneal blood collection. CT scan is invaluable for diagnosis and treatment planning because it can check the severity of the cirrhosis and locate the tumor.

Rupture of a hepatic metastasis frequently results in massive hemoperitoneum, which may be a terminal event [21]. For these patients, it is therefore crucial to control the bleeding and restore the blood pressure as soon as possible. Transcatheter hepatic arterial embolization (TAE) may seem to be ideal for these patients, since it avoids general anesthesia to stop the bleeding without operation [22], but it also has some demerits, including recurrent bleeding and liver failure [22,23], peritoneal abscess [24], and implanted metastases [25,26]. Furthermore, long-term results are poor if it is used as a sole treatment approach [27], and it is not applicable when the main trunk of the portal vein is obstructed. In addition, a two-stage surgical operation (that is, TAE followed by liver resection) would inevitably increase the hospital stay and the cost. For our patients, one-stage hepatectomy is still a good treatment method for those who are stable hemodynamically and has a reasonable liver function as well as the lesion is peripherally located.

## Conclusions

Small cell neuroendocrine carcinoma of the maxillary sinus is very rare and has a poor prognosis. It may cause spontaneous metastatic rupture which resulted in fatal hemorrhage. Emergency surgery is effective but the outcome is still unsatisfactory.

## Consent

Written informed consent was obtained from the patient’s son for publication of this report and any accompanying images.

## Competing interests

The authors declare that they have no competing interests. No benefits in any form have been received or will be received from a commercial party related directly or indirectly to the subject of this article.

## Authors’ contributions

Y-fD and FZ participated in the clinical management of the patient and wrote the manuscript. YT and BY carried out the pathological examination. Y-fD and FZ were involved in the final editing. All authors approved the final manuscript.
